# Laparoscopic vs open colorectal surgery: Economic and clinical outcomes in the Brazilian healthcare

**DOI:** 10.1097/MD.0000000000022718

**Published:** 2020-10-16

**Authors:** Ulysses Ribeiro, Daiane Oliveira Tayar, Rodrigo Antonini Ribeiro, Priscila Andrade, Silvio Mauro Junqueira

**Affiliations:** a Department of Digestive Surgery, Faculdade de Medicina da Universidade de São Paulo; b Department of Health Economics and Market Access, Johnson & Johnson Medical, São Paulo; c Department of Health Economics, HTAnalyze Consulting, Porto Alegre, Rio Grande do Sul, Brazil.

**Keywords:** colorectal surgery, cost analysis, laparoscopy

## Abstract

Laparoscopic surgery has become the preferred surgical approach of several colorectal conditions. However, the economic results of this are quite controversial. The degree of adoption of laparoscopic technology, as well as the aptitude of the surgeons, can have an influence not only in the clinical outcomes but also in the total procedure cost. The aim of this study was to evaluate the clinical and economic outcomes of laparoscopic colorectal surgeries, compared to open procedures in Brazil.

All patients who underwent elective colorectal surgeries between January 2012 and December 2013 were eligible to the retrospective cohort. The considered follow-up period was within 30 days from the index procedure. The outcomes evaluated were the length of stay, blood transfusion, intensive care unit admission, in-hospital mortality, use of antibiotics, the development of anastomotic leakage, readmission, and the total hospital costs including re-admissions.

Two hundred eighty patients, who met the eligibility criteria, were included in the analysis. Patients in the laparoscopic group had a shorter length of stay in comparison with the open group (6.02 ± 3.86 vs 9.86 ± 16.27, *P* < .001). There were no significant differences in other clinical outcomes between the 2 groups. The total costs were similar between the 2 groups, in the multivariate analysis (generalized linear model ratio of means 1.20, *P* = .074). The cost predictors were the cancer diagnosis and age.

Laparoscopic colorectal surgery presents a 17% decrease in the duration of the hospital stay without increasing the total hospitalization costs. The factors associated with increased hospital costs were age and the diagnosis of cancer.

## 1 Introduction

Laparoscopic surgery has become the preferred method in the surgical treatment of several colorectal diseases. Many studies, including randomized clinical trials, have shown that laparoscopic access is both effective and safe, with better short-term results in patient recovery compared to the open surgical approach.^[1–14]^

Regarding long-term clinical outcomes, studies have shown that there are no differences between laparoscopic or open surgery in cancer patients, or that laparoscopic surgery is superior to open surgery in the disease-free survival rate of colonic resections.^[1,9,14–18]^

Regarding the economic outcomes, the evidence is rather controversial. Although a few publications have shown that laparoscopic colorectal surgery is more costly, despite the better short-term clinical outcomes,^[19–22]^ others have demonstrated that laparoscopy may be either cost-neutral or yield savings from the perspective of hospitals and the payers.^[4,10,11,23–26]^

The difference in the results might be explained by the impact of the cost of the device in the total hospital cost, according to the healthcare system and health technology incorporation characteristics of each country.^[27]^ Besides, laparoscopy adoption in a country, as well as the degree of aptitude of the surgeons can influence the clinical outcomes and, consequently, the total procedure cost.^[20,28–30]^ Some publications have shown that after the learning curve period, laparoscopic surgery may either be cost-neutral, or cost-saving compared to the open surgical approach.^[31]^

Thus, the objective of this study was to evaluate the clinical and economic outcomes of laparoscopic colorectal surgeries, compared to open procedures, in the Brazilian private healthcare system according to the perspective of the payers and providers.

## 2 Methods

### 2.1 Study design

The study was a retrospective cohort. The dataset used for the analyses was a hospital patients’ billings database (reimbursement information) from Orizon. The database contains information about the transaction between health insurance plans and hospitals from the Brazilian private healthcare system, including all the billed items during hospital admissions. It also contains information from ambulatory service transactions. About 12 million patients – who represent around 25% of patients in the Brazilian private healthcare system – are included in Orizon's registries.

All patients who were admitted between January 2012 and December 2013 who underwent rectum or sigmoid colon procedures (hereafter referred to as colorectal surgeries) were considered potentially eligible. The TUSS codes used to determine the procedures were: 31003559 – retossigmoidectomia abdominal (abdominal rectosigmoidectomy) and 31003796 – retossigmoidectomia abdominal por videolaparoscopia (laparoscopic abdominal rectosigmoidectomy). The eligible patients had their procedure in multiple location across different states in Brazil, but due to confidentiality rules, the location of the patients was not described in the dataset.

The following eligibility criteria were used for the inclusion of patients:

- Availability of age, gender, international classification of diseases (ICD) code information and surgical approach (laparoscopic vs open);
- The procedure should be elective (urgent surgeries were excluded).
- The colorectal surgery must have been performed in a maximum of 3 days after hospital admission;

The outcomes of perioperative procedures and readmission for up to 30 days were analyzed. As all the analyzed data were obtained from the administrative claims data of the routine care of the patients and were made anonymous by the researchers, no informed consent was required. Lastly, as there was no possibility of any harm arising as a result of the conduct of this research, no Ethical Review Board approval was required.

### 2.2 Study variables

The Patients were grouped according to surgical approach (laparoscopic vs open). The following variables were evaluated for each patient: age, gender, ICD code for the admission related to the surgery: cancer status, use of chemotherapy and radiotherapy, extent of the surgery (multivisceral resection vs simple procedures), need for perioperative blood transfusion, length of stay (LOS), intensive care unit (ICU) admission, 30-day readmission, the use of antibiotics, mortality, the occurrence of anastomotic leakage, and hospital costs.

Surgical treatment involving only the rectum or sigmoid colon with or without lymph nodes resection and with or without colostomy associated, were classified as simple procedures. If an additional procedure including hysterectomy, oophorectomy, or cystectomy were billed on the same day of the colorectal procedure, the surgery was classified as a multivisceral resection.

Perioperative transfusion was defined as the use of a concentrate of red blood cells between the date of surgery and the following 30 days. Readmissions were defined whenever a patient had billed items for at least 2 consecutive days. Only an emergency room visit was not considered readmission.

The rate of the use of postoperative antibiotics was defined as a postoperative exposure (up to 30 days from index surgery) to antimicrobial drugs, which suggested a therapeutic use – whenever the utilization was suggestive of prophylaxis, it was not considered. The criteria used were: prescriptions for at least 7 days and use of 1 of the following antimicrobials: carbapenems, 3^rd^ and 4^th^ generation cephalosporins, vancomycin, aminoglycosides, piperacillin, and a combination of quinolones with either clindamycin or metronidazole.

The definition of anastomotic Leakage occurrence was the result of the simultaneous presence of the following conditions:

1. The patient had an infection under investigation, where the proxy used was the billing of a postoperative blood culture exam;
2. There was at least 1 postoperative imaging study, such as an abdominal X-ray, computerized tomography or another abdominal exam with an intravenous contrast.
3. A second surgical abdominal procedure or image-guided percutaneous drainage must have been undertaken up to 30 days after the index surgery.

Hospital costs were categorized as index admission costs, which included all billed items during the index surgery admission, and 30-day readmissions costs, which included all the billed items from any other hospital admission initiated within 30 days of the index surgery. In both cases, we did not use a termination date. If the index admission or a readmission began in less than 30 days after the index surgery and lasted for example 4 months, all expenditures associated with this admission were included. The total patients’ costs were also calculated - by summing the costs of the index admission and 30-day readmission.

### 2.3 Statistical analysis

Categorical variables are presented as absolute values and proportions, and continuous variables are presented as the mean and standard deviation. Comparison between the laparoscopic versus open surgery groups employed the Student Fisher exact test for categorical variables and the Student *t* test for continuous variables with normal distribution. Considering that both costs and LOS, the 2 main continuous outcomes of this study, usually present a gamma distribution, we also employed univariate generalized linear model (GLM) with the gamma distribution for these analyses.

Multivariate analyses used Poisson regression for dichotomous outcomes (which was chosen as some of the outcomes had an incidence much larger than 10%, contraindicating logistic regression) and GLMs for costs and LOS. Variables with a *P* value of .10 or less in the univariate analyses were selected to enter the multivariate analyses. In all analyses, a *P* value smaller than .05 was considered statistically significant. SPSS (Statistical Package for Social Sciences, version 20.0, Chicago, IL, USA) was used for the analyses.

## 3 Results

**Figure 1:**
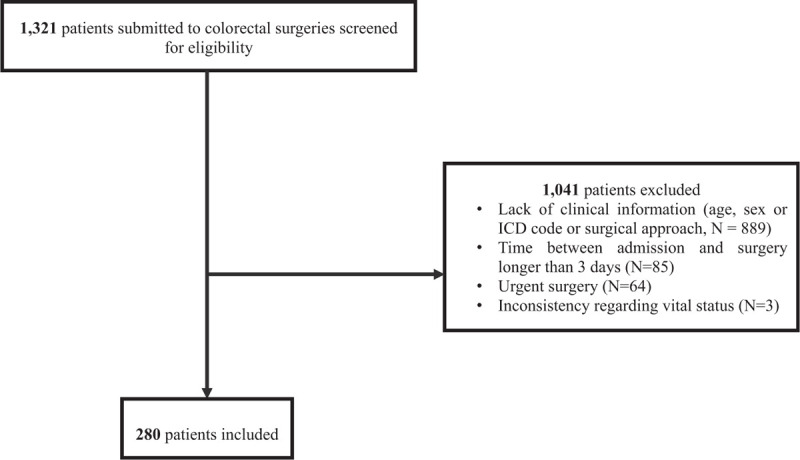
Flowchart of the patient selection for the study.

Among 1.321 potentially eligible patients, 280 fulfilled the eligibility criteria and were included in the analyses (Fig. 1). The group of patients operated laparoscopically were younger (mean age of 47 years, vs 53 in the open surgical group, *P* = .001), and had a smaller proportion of cancer diagnosis (17.2% vs 41.5% in the open surgical group, *P* < .001), as shown in Table 1.

**Table 1:**
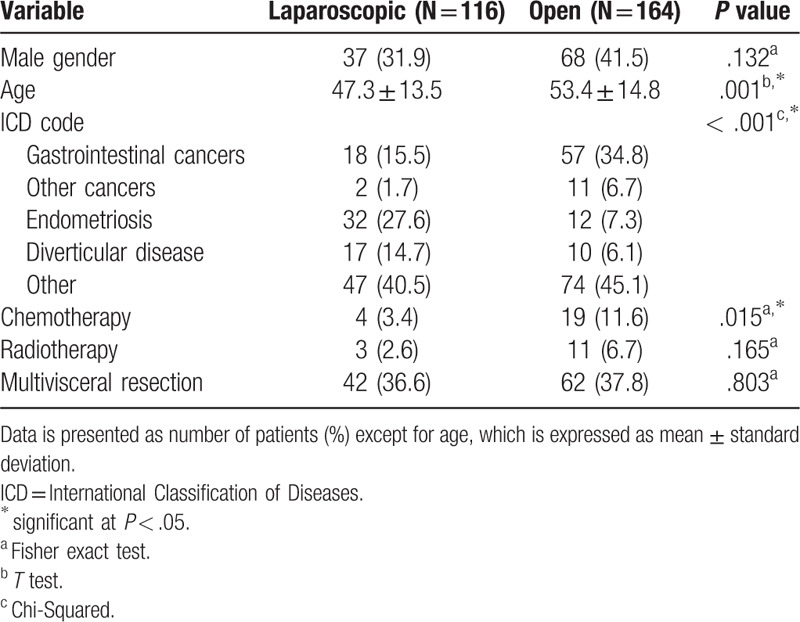
Demographics and perioperative data of the study cohort according to surgical approach.

The proportion of male patients and multivisceral resections did not show a statistically significant difference. Although the comparison of the 2 groups showed a higher proportion of patients receiving chemotherapy in the open surgery group, the proportion of such patients considering only the ones with cancer in each group did not reach statistically significant difference (15.0% in the laparoscopic vs 27.9%, *P* = .379).

In the univariate analysis evaluating clinical outcomes (Table 2), the proportion of patients with blood transfusion requirement and ICU admissions was smaller in the laparoscopic group. In the multivariate analysis, however, these outcomes did not reach the statistically significant differences (adjusted relative risk [RR] for transfusion need = 0.54, 95% confidence interval [CI] 0.27–1.05, adjusted RR for ICU admission = 0.84, 95% CI 0.65–1.09).

**Table 2:**
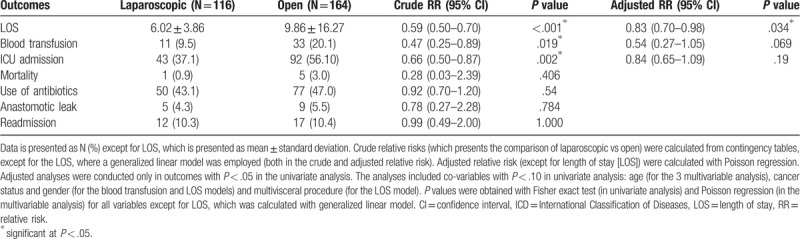
Clinical outcomes of 280 patients according to surgical approach.

Patients in the laparoscopic surgery group had a shorter LOS, which was reduced by 17% (95% CI: 2%–30%) in the analysis adjusted for age, gender, cancer status, and multivisceral resection. The incidence of anastomotic leak, readmissions, use of antibiotics and the mortality rate did not differ between the 2 groups.

The mean index admission costs (presented in Table 3) were lower in the laparoscopic group (R$ 32,915, vs R$ 41,652 in the open surgery group, *P* = .024), as well as total costs, where 30-day readmissions were also computed (R$ 35,424 in the laparoscopic vs R$ 44,461 in the open surgery group, *P* = .026).

**Table 3:**
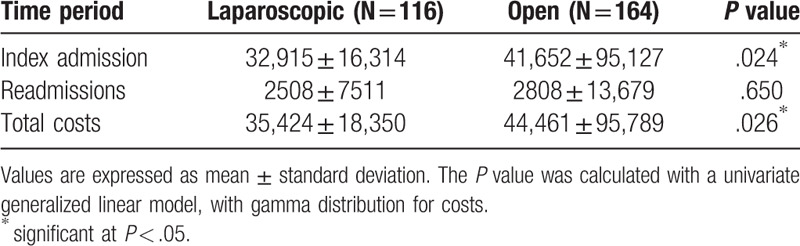
Cost outcomes (Brazilian R$) according to surgical approach.

Conversely, in the GLM multivariate analysis of total costs (Table 4), with adjustments for age, gender, and cancer status, the means’ ratio was not statistically significant: 1.20, 95% CI 0.98–1.46 (*P* = .074). Results for the index admission costs were very similar (data not being shown). The predictors that remained statistically significant in the multivariate analyses were the status of cancer (GLM ratio of means = 1.84, 95% CI 1.48–2.28) and the age (GLM ratio of means = 1.02, 95% CI 1.01–1.02).

**Table 4:**
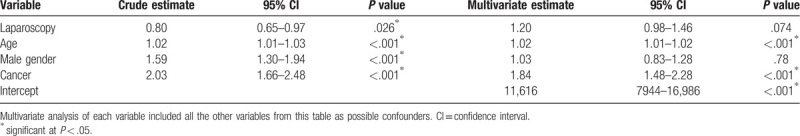
Generalized linear model for total costs.

## 4 Discussion

In this study, which compared economic and clinical outcomes between laparoscopic and open colorectal surgery in a sample of patients from the Brazilian private healthcare system, the main difference between the 2 groups was the LOS. After adjustment for age, gender, cancer status, and multivisceral resection in the multivariate analysis, the LOS was 17% shorter (95% CI: 2%–30%) in the laparoscopic group. This result is comparable to those found in the literature that show that laparoscopic rectal surgeries present a shorter hospitalization period compared to open surgeries.^[1,2,4,7,9,11–14,21,24]^

Regarding the other clinical outcomes in the current study, patients had a lower need of blood transfusion and a smaller ICU admission rate in the univariate analysis. However, these associations did not maintain statistical significance in the multivariate analysis. The requirement for blood transfusions, which showed a 53% decrease in univariate analysis (95% CI: 11%–75%), lost it significance in the analysis adjusted for age, gender, and the cancer status (RR = 0.54, 95% CI: 0.27–1.05, *P* = .069). The ICU admission rates, which were reduced by 34% in the univariate analysis, lost its significance in the analysis adjusted for age (RR = 0.84, 95% CI: 0.65–1.09, *P* = .19). It is likely that the analysis of clinical outcomes suffered from insufficient statistical power, especially for mortality, where the number of events was small.

The results regarding index admission and total expenses (which included 30-day readmissions) demonstrated that the laparoscopic approach is not more costly than open surgery. In fact, in the univariate analysis, laparoscopic surgery was associated with a lower cost when compared to the open approach and presented a lower standard deviation, which could suggest that laparoscopy has a greater predictability of total costs in comparison with the open surgery (35,424 ± 18,350 vs 44,461 ± 95,789, *P* = .026). However, this comparison lost statistical significance in the multivariate GLM. Age and cancer diagnosis were identified as independent risk factors to increase the total hospital costs in colorectal procedures (multivariate estimate: 1.02, 95% CI: 1.01–1.02, *P* < .001) and (multivariate estimate: 1.84, 95% CI: 1.48–2.28, *P* < .001), respectively.

This study shows that, in the Brazilian private healthcare system scenario, laparoscopic colorectal surgery is not more expensive than the open procedure. This cost comparison has a great variability in the literature, which suggests that the impact of the cost of medical devices in the total hospital costs, can be the leading cause to determine if laparoscopy is cost-saving or expensive.^[19,21,22,32]^

The cost balance between the 2 surgical approaches occurs when the reduction of postoperative costs, resulting from better short-term clinical outcomes, compensate higher intraoperative costs, which can result in cost neutrality or even saving costs. In addition to the present study, other studies in the literature have found cost neutrality between the open and laparoscopic approach.^[4,10,33]^

In a study with rectal cancer patients, which used propensity score matching, laparoscopy had intraoperative costs which were 21% higher than the open approach (*P* < .001). However, non-surgical costs, including laboratory, radiology, and nursing post-operative care were lower in the laparoscopic group. The total costs were 7% lower in the laparoscopic group, although this difference was not significant.^[4]^

It should be noted that the studies that evaluated the costs of open and laparoscopic colorectal procedures are mostly focused on rectal cancer. The present study aimed to evaluate all diseases that required colorectal surgical intervention, including benign diseases, showing that laparoscopic surgery may be cost-neutral in comparison to the open surgery in a wider population as well.

This comparative evaluation of the costs and clinical outcomes from a hospital and payer perspective is unprecedented in the Brazilian private healthcare system, and its importance lies in the fact that the budgetary constraints imposed on the private health systems have been aggravated by the medical scenario and the scenario of the country's economic recession.

Some limitations in our study must be acknowledged.

The database used for all analyses was based on the patients’ billing information. This approach presents the risk of bias of other retrospective studies, and the limitations secondary to the lack of individual clinical patient information. A better adjustment in the multivariate models would probably be achieved with more detailed clinical information.

The exclusion of the majority of the original sample (secondary to eligibility criteria) can lead to selection bias and compromise the generalizability of the study. In this regard, the high proportion of patients with an endometriosis ICD code is noteworthy. Although this disease is seldom presented as a frequent motive for colorectal surgeries, its rate can be substantial, as seen in a national colorectal surgeries database from France.^[34]^ One possible explanation for the high proportion of endometriosis in this study is that, considering that the disease is fairly uncommon, an indication for colorectal surgeries, the physician might have informed this ICD code more accurately than other diseases, in order to receive approval by the health insurance plans. Since our inclusion criteria demanded information about the ICD code, the high proportion of endometriosis in the sample might be the consequence of better filling of this information by physicians.

Lastly, some of the 95% CI from the estimates were substantially wide (except in the costs GLM), suggesting that a larger sample would be important to confirm some of our findings.

## 5 Conclusion

The Real-World Evidence of the Brazilian Healthcare System shows that laparoscopic colorectal surgery presents a 17% decrease in the hospital stay time without increasing the total hospitalization costs. Factors associated with increased hospital costs were age and the diagnosis of cancer. These findings suggest that the laparoscopic procedure may be considered the preferred procedure in patients submitted to colorectal surgery, regardless of the type of pathology, whenever there are no contraindications. The study also concludes that the laparoscopic method can be economically sustainable even in developing countries that present budgetary constraints.

## Author contributions

All authors had full access to all the data in the study and assumed responsibility for all aspects of the work, ensuring that questions related to the accuracy or integrity of any part of the work were appropriately investigated and resolved. Furthermore, all authors were accountable for the parts of the work they did, able to identify which co-authors were responsible for specific other parts of the work and confident in the integrity of the contributions of their co-authors. Ulysses Ribeiro Jr affirms that this manuscript is an honest, accurate, and transparent account of the study being reported; that no important aspects of the study have been omitted; and that any discrepancies from the study as planned (and, if relevant, registered) have been explained. Ulysses Ribeiro Jr, Daiane Oliveira Tayar, Rodrigo Antonini Ribeiro, Priscila Andrade, and Silvio Mauro Junqueira Jr had substantial contribution to the conception and design of the work; Ulysses Ribeiro Jr, Daiane Oliveira Tayar, and Rodrigo Antonini Ribeiro had substantial contribution in the data acquisition and/or analysis and interpretation of the data of the work; Daiane Oliveira Tayar and Rodrigo Antonini Ribeiro drafted the manuscript; Ulysses Ribeiro Jr, Daiane Oliveira Tayar, Priscila Andrade, and Silvio Mauro Junqueira Jr reviewed the article critically for important intellectual content; all authors provided the final approval of the version to be published.

**Conceptualization:** Ulysses Ribeiro Jr, Daiane Oliveira Tayar.

**Data curation:** Ulysses Ribeiro Jr, Daiane Oliveira Tayar, Rodrigo Antonini Ribeiro.

**Formal analysis:** Rodrigo Antonini Ribeiro.

**Funding acquisition:** Priscila Andrade, Silvio Mauro Junqueira Jr.

**Investigation:** Ulysses Ribeiro Jr.

**Methodology:** Ulysses Ribeiro Jr, Daiane Oliveira Tayar, Rodrigo Antonini Ribeiro, Priscila Andrade, Silvio Mauro Junqueira Jr.

**Project administration:** Priscila Andrade, Silvio Mauro Junqueira Jr.

**Resources:** Priscila Andrade, Silvio Mauro Junqueira Jr.

**Software:** Rodrigo Antonini Ribeiro.

**Supervision:** Ulysses Ribeiro Jr, Daiane Oliveira Tayar, Priscila Andrade, Silvio Mauro Junqueira Jr.

**Validation:** Ulysses Ribeiro Jr, Daiane Oliveira Tayar, Rodrigo Antonini Ribeiro, Priscila Andrade, Silvio Mauro Junqueira Jr.

**Visualization:** Ulysses Ribeiro Jr, Daiane Oliveira Tayar, Rodrigo Antonini Ribeiro, Priscila Andrade, Silvio Mauro Junqueira Jr.

**Writing – original draft:** Daiane Oliveira Tayar, Rodrigo Antonini Ribeiro.

**Writing – review & editing:** Ulysses Ribeiro Jr, Daiane Oliveira Tayar, Rodrigo Antonini Ribeiro, Priscila Andrade, Silvio Mauro Junqueira Jr.

## References

[1] Chen K Cao G Chen B Laparoscopic versus open surgery for rectal cancer: a meta-analysis of classic randomized controlled trials and high-quality nonrandomized studies in the last 5 years. Int J Surg 2017;39:1–0. 28087370 10.1016/j.ijsu.2016.12.123

[2] de’Angelis N Landi F Vitali G Multicentre propensity score-matched analysis of laparoscopic versus open surgery for T4 rectal cancer. Surg Endosc 2016;31:3106–21. 27826780 10.1007/s00464-016-5332-9

[3] Devoto L Celentano V Cohen R Colorectal cancer surgery in the very elderly patient: a systematic review of laparoscopic versus open colorectal resection. Int J Colorectal Dis 2017;32:1237–42. 28667498 10.1007/s00384-017-2848-y

[4] Hayashi H Ozaki N Ogawa K Assessing the economic advantage of laparoscopic vs. open approaches for colorectal cancer by a propensity score matching analysis. Surg Today 2017;48:439–48. 29110090 10.1007/s00595-017-1606-7

[5] Inada R Yamamoto S Oshiro T A case-matched comparison of the short-term outcomes between laparoscopic and open abdominoperineal resection for rectal cancer. Surg Today 2013;44:640–5. 23670040 10.1007/s00595-013-0611-8

[6] Kang S Park J Jeong S Open versus laparoscopic surgery for mid or low rectal cancer after neoadjuvant chemoradiotherapy (COREAN trial): short-term outcomes of an open-label randomised controlled trial. Lancet Oncol 2010;11:637–45. 20610322 10.1016/S1470-2045(10)70131-5

[7] Małczak P Mizera M Torbicz G Is the laparoscopic approach for rectal cancer superior to open surgery? A systematic review and meta-analysis on short-term surgical outcomes. Wideochir Inne Tech Maloinwazyjne 2018;13:129–40. 30002744 10.5114/wiitm.2018.75845 PMC6041579

[8] Numata M Sawazaki S Morita J Comparison of laparoscopic and open surgery for colorectal cancer in patients with severe comorbidities. Anticancer Res 2018;38:963–7. 29374728 10.21873/anticanres.12310

[9] Ohtani H Tamamori Y Azuma T A meta-analysis of the short- and long-term results of randomized controlled trials that compared laparoscopy-assisted and conventional open surgery for rectal cancer. J Gastrointest Surg 2011;15:1375–85. 21557014 10.1007/s11605-011-1547-1

[10] Silva-Velazco J Dietz DW Stocchi L Considering value in rectal cancer surgery: an analysis of costs and outcomes based on the open, laparoscopic, and robotic approach for proctectomy. Ann Surg 2017;265:960–8. 27232247 10.1097/SLA.0000000000001815

[11] Thompson BS Coory MD Gordon LG Cost savings for elective laparoscopic resection compared with open resection for colorectal cancer in a region of high uptake. Surg Endosc 2014;28:1515–21. 24337191 10.1007/s00464-013-3345-1

[12] Trastulli S Cirocchi R Listorti C Laparoscopic vs open resection for rectal cancer: a meta-analysis of randomized clinical trials. Colorectal Dis 2012;14:e277–96. 22330061 10.1111/j.1463-1318.2012.02985.x

[13] van der Pas MH Haglind E Cuesta MA Laparoscopic versus open surgery for rectal cancer (COLOR II): short-term outcomes of a randomised, phase 3 trial. Lancet Oncol 2013;14:210–8. 23395398 10.1016/S1470-2045(13)70016-0

[14] Vennix S Pelzers L Bouvy N Laparoscopic versus open total mesorectal excision for rectal cancer. Cochrane Database Syst Rev 2014;(4):CD005200. 24737031 10.1002/14651858.CD005200.pub3 PMC10875406

[15] Draeger T Völkel V Gerken M Long-term oncologic outcomes after laparoscopic versus open rectal cancer resection: a high-quality population-based analysis in a Southern German district. Surg Endosc 2018;32:4096–104. 29611044 10.1007/s00464-018-6148-6 PMC6132875

[16] Ishibe A Ota M Fujii S Midterm follow-up of a randomized trial of open surgery versus laparoscopic surgery in elderly patients with colorectal cancer. Surg Endosc 2017;31:3890–7. 28205033 10.1007/s00464-017-5418-z

[17] Jayne DG Thorpe HC Copeland J Five-year follow-up of the Medical Research Council CLASICC trial of laparoscopically assisted versus open surgery for colorectal cancer. Br J Surg 2010;97:1638–45. 20629110 10.1002/bjs.7160

[18] Pędziwiatr M Małczak P Mizera M There is no difference in outcome between laparoscopic and open surgery for rectal cancer: a systematic review and meta-analysis on short- and long-term oncologic outcomes. Tech Coloproctol 2017;21:595–604. 28795243 10.1007/s10151-017-1662-4 PMC5602007

[19] Gehrman J Bjorholt I Angenete E Health economic analysis of costs of laparoscopic and open surgery for rectal cancer within a randomized trial (COLOR II). Surg Endosc 2017;31:1225–34. 27422249 10.1007/s00464-016-5096-2 PMC5315718

[20] Son GM Kim JG Lee JC Multidimensional analysis of the learning curve for laparoscopic rectal cancer surgery. J Laparoendosc Adv Surg Tech A 2010;20:609–17. 20701545 10.1089/lap.2010.0007

[21] Bo D Bo H Yonghong D Comparison between laparoscopic and open total mesorectal excision in the treatment of rectal cancer. Cancer Res Clin 2016;28:679–82.

[22] Wu Y Sun X Qi J Comparative study of short- and long-term outcomes of laparoscopic-assisted versus open rectal cancer resection during and after the learning curve period. Medicine 2017;96:1–7. 10.1097/MD.0000000000006909 PMC5428641 28489807

[23] Berto P Lopatriello S Aiello A Cost of laparoscopy and laparotomy in the surgical treatment of colorectal cancer. Surg Endosc 2012;26:1444–53. 22179444 10.1007/s00464-011-2053-y

[24] Keller DS Champagne BJ Reynolds HL Jr Cost-effectiveness of laparoscopy in rectal cancer. Dis Colon Rectum 2014;57:564–9. 24819095 10.1097/DCR.0b013e3182a73244

[25] Ramji KM Cleghorn MC Josse JM Comparison of clinical and economic outcomes between robotic, laparoscopic, and open rectal cancer surgery: early experience at a tertiary care center. Surg Endosc 2016;30:1337–43. 26173546 10.1007/s00464-015-4390-8

[26] Oliveira Tayar D Ribeiro U Jr Cecconello I Propensity score matching comparison of laparoscopic versus open surgery for rectal cancer in a middle-income country: short-term outcomes and cost analysis. Clinicoecon Outcomes Res 2018;10:521–7. 30254479 10.2147/CEOR.S173718 PMC6140693

[27] Callea G Armeni P Marsilio M The impact of HTA and procurement practices on the selection and prices of medical devices. Soc Sci Med 2017;174:89–95. 28013109 10.1016/j.socscimed.2016.11.038

[28] Ito M Sugito M Kobayashi A Influence of learning curve on short-term results after laparoscopic resection for rectal cancer. Surg Endosc 2009;23:403–8. 18401643 10.1007/s00464-008-9912-1

[29] Keller DS Qiu J Senagore AJ Predicting opportunities to increase utilization of laparoscopy for rectal cancer. Surg Endosc 2018;32:1556–63. 28917020 10.1007/s00464-017-5844-y

[30] Li GX Yan HT Yu J Learning curve of laparoscopic resection for rectal cancer. Nan Fang Yi Ke Da Xue Xue Bao 2006;26:535–8. 16624777

[31] Park JS Choi GS Kim SH Multicenter analysis of risk factors for anastomotic leakage after laparoscopic rectal cancer excision: the Korean laparoscopic colorectal surgery study group. Ann Surg 2013;257:665–71. 23333881 10.1097/SLA.0b013e31827b8ed9

[32] Braga M Vignali A Zuliani W Laparoscopic versus open colorectal surgery: cost-benefit analysis in a single-center randomized trial. Ann Surg 2005;242:890–6. 16327499 10.1097/01.sla.0000189573.23744.59 PMC1409893

[33] Norwood MG Stephens JH Hewett PJ The nursing and financial implications of laparoscopic colorectal surgery: data from a randomized controlled trial. Colorectal Dis 2011;13:1303–7. 20955511 10.1111/j.1463-1318.2010.02446.x

[34] Roman H A national snapshot of the surgical management of deep infiltrating endometriosis of the rectum and colon in France in 2015: a multicenter series of 1135 cases. J Gynecol Obstet Hum Reprod 2017;46:159–65. 28403973 10.1016/j.jogoh.2016.09.004

